# The Berlin Treatment Algorithm: recommendations for tailored innovative therapeutic strategies for multiple sclerosis-related fatigue

**DOI:** 10.1186/s13167-016-0073-3

**Published:** 2016-11-24

**Authors:** Christian Veauthier, Helge Hasselmann, Stefan M. Gold, Friedemann Paul

**Affiliations:** 1 Interdisciplinary Center for Sleep Medicine, Charité – Universitätsmedizin Berlin, Charitéplatz 1, 10117 Berlin, Germany; 2 NeuroCure Clinical Research Center, Charité – Universitätsmedizin Berlin, Charitéplatz 1, 10117 Berlin, Germany; 3 Department of Psychiatry and Psychotherapy, Charité – Universitätsmedizin Berlin, Hindenburgdamm 30, 12203 Berlin, Germany; 4 Institute of Neuroimmunology and Multiple Sclerosis (INIMS), Center for Molecular Neurobiology (ZMNH), University Medical Center Hamburg-Eppendorf, 20251 Hamburg, Germany; 5 Clinical and Experimental Multiple Sclerosis Research Center, Department of Neurology, Charité – Universitätsmedizin Berlin, 10117 Berlin, Germany; 6 Experimental and Clinical Research Center, Max Delbrück Center for Molecular Medicine and Charité – Universitätsmedizin Berlin, Berlin, Germany

**Keywords:** Polysomnography, Restless legs syndrome, Personalized medicine, Resistance training, Cognitive behavioral therapy, Obstructive sleep apnea, Patient stratification, Depression, Tiredness, Fatigue, Multiple sclerosis

## Abstract

More than 80% of multiple sclerosis (MS) patients suffer from fatigue. Despite this, there are few therapeutic options and evidence-based pharmacological treatments are lacking. The associated societal burden is substantial (MS fatigue is a major reason for part-time employment or early retirement), and at least one out of four MS patients view fatigue as the most burdensome symptom of their disease. The mechanisms underlying MS-related fatigue are poorly understood, and objective criteria for distinguishing and evaluating levels of fatigue and tiredness have not yet been developed. A further complication is that both symptoms may also be unspecific indicators of many other diseases (including depression, sleep disorders, anemia, renal failure, liver diseases, chronic obstructive pulmonary disease, drug side effects, recent MS relapses, infections, nocturia, cancer, thyroid hypofunction, lack of physical exercise). This paper reviews current treatment options of MS-related fatigue in order to establish an individualized therapeutic strategy that factors in existing comorbid disorders. To ensure that such a strategy can also be easily and widely implemented, a comprehensive approach is needed, which ideally takes into account all other possible causes and which is moreover cost efficient. Using a diagnostic interview, depressive disorders, sleep disorders and side effects of the medication should be identified and addressed. All MS patients suffering from fatigue should fill out the Modified Fatigue Impact Scale, Epworth Sleepiness Scale, the Beck Depression Inventory (or a similar depression scale), and the Pittsburgh Sleep Quality Index (or the Insomnia Severity Index). In some patients, polygraphic or polysomnographic investigations should be performed. The treatment of underlying sleep disorders, drug therapy with alfacalcidol or fampridine, exercise therapy, and cognitive behavioral therapy-based interventions may be effective against MS-related fatigue. The objectives of this article are to identify the reasons for fatigue in patients suffering from multiple sclerosis and to introduce individually tailored treatment approaches. Moreover, this paper focuses on current knowledge about MS-related fatigue in relation to brain atrophy and lesions, cognition, disease course, and other findings in an attempt to identify future research directions.

## Background

Multiple sclerosis (MS) is a chronic inflammatory and neurodegenerative autoimmune disease of the central nervous system with multifactorial etiopathogenesis, which predominantly affects young adults and women [1–3]. At least one out of four MS patients views fatigue as the most burdensome symptom of their illness, and the majority of MS patients (more than 80%) suffer from fatigue [4]. Fatigue may occur at any stage of the disease and can even precede MS onset by several years [5]. Fatigue is a major reason for early retirement, reduced employment, and poor quality of life in people with MS [6–8].

The 1998 clinical practice guidelines recommended by the Consortium of Multiple Sclerosis Centers (the so-called MS Council, a consensus group of 22 North American associations including the American Academy of Neurology, the Consortium of Multiple Sclerosis Centers, and the US National Multiple Sclerosis Society) have defined MS-related fatigue as “a subjective lack of physical and/or mental energy that is perceived by the individual or caregiver to interfere with usual and desired activities” [9]. According to this definition, fatigue not only is a symptom but also has a major impact on daily activities.

The precise mechanisms of MS-related fatigue are not known, and several non-MS-related possible reasons for fatigue and tiredness should be taken into account [2]. This paper reviews current data about MS-related fatigue and neuroanatomical findings, cognitive impairment, depression, disease-modifying therapies, and disease course, on the one hand, and major confounders and other (symptomatic) reasons for fatigue and tiredness, on the other. Furthermore, the aim of this paper is to provide physicians with a clinically applicable treatment algorithm for MS-related fatigue. This review is an updated and extended version of a recently published article in German [10].

## Assessment and diagnostics

### Fatigue questionnaires

Several scales are available for measuring fatigue. The first published fatigue scale was the unidimensional Fatigue Severity Scale (FSS) in 1989 identifying the existence and severity of fatigue [11]. Nine years later, the MS Council recommended the multidimensional Modified Fatigue Impact Scale (MFIS, 21 items), measuring the impact of fatigue on cognitive functioning and psychosocial as well as physical domains [9]. The MFIS is an abbreviated version of the *Fatigue Impact Scale* (FIS, 40 items) [12]. The FIS itself is a more concise version of the 138 items listed by the “Multiple Sclerosis Quality-of-Life Inventory” (MSQLI) [13]. The FSS and the MFIS are not specific for MS-related fatigue and show an overlap with depression and tiredness due to sleep disorders [2, 11, 14]. Most studies used a MFIS cutoff of 38 or 45 [15–17]. For details about the psychometric data and validation of these questionnaires, please see a recent review [2]. Finally, in 2009, the Fatigue Scale for Motor and Cognitive Functions (FSMC) was introduced [18].

### MS fatigue and tiredness

Unlike sleepiness, mental (cognitive) fatigue and tiredness cannot be measured by polysomnography or electroencephalography. Both can only be assessed indirectly by neuropsychological investigations or vigorimeter testing and handgrip performance [19].

Moreover, outside the specific context of MS, patients suffering from sleep disorders who were consecutively admitted to a sleep laboratory also showed high mean MFIS values (untreated sleep apnea 32.5, periodic limb movement disorder and restless legs syndrome 44.1, insomnia 33.5) [14]. The same was found for the FSS. Compared with men and older patients, the MFIS and FSS values were higher in women and in young patients. This is of particular importance because young women are typically overrepresented among MS patients.

In summary, no objective diagnostic criteria for distinguishing between MS- and non-MS-related tiredness and fatigue exist. For this reason, other possible underlying causes of these symptoms have to be taken into account.

### MS-related fatigue versus sleepiness

MS-related fatigue and tiredness can clinically and objectively (by polysomnography and by the multiple sleep latency test) be distinguished from sleepiness: Sleepiness is defined by the propensity to fall asleep, often associated with an effort to avoid sleeping and is generally caused by sleep disorders or by disturbances of the circadian rhythm [20, 21]. Therefore, screening for sleepiness is very important in fatigued MS patients because sleepiness is an important indicator for an underlying sleep disorder [22, 23]. All fatigued patients should be asked about sleepiness and fill in the Epworth Sleepiness Scale (ESS). ESS values equal or greater than 10 indicate excessive daytime sleepiness (EDS), and in this case, patients should undergo polygraphy or polysomnography [20].

However, in sleep disorders outside the context of MS (in patients suffering from sleep disorders in the general population who are admitted to a sleep laboratory), fatigue and sleepiness can occur independently or simultaneously [24, 25]. The majority of them, almost two thirds, report pathological fatigue *without* sleepiness and almost one fifth fatigue *and* sleepiness. However, only a small number of patients with sleep disorders (4%) suffer from sleepiness and do not feel fatigued (13% experienced neither fatigue nor sleepiness) [25]. This means that it is very important to ask MS patients about sleepiness, because sleepiness indicates sleep problems and is an argument against MS-related fatigue. However, these data show clearly that the lack of sleepiness is not an argument against the presence of sleep disorders: although sleepiness is more specific to an underlying sleep disorder, fatigue remains the most common symptom of an underlying sleep disorder.

### Fatigability and motor fatigue

We have to distinguish between sustained perceived fatigue from load-dependent fatigability, on the one hand, and impaired performance during a motor task and an increased subjective experience of fatigue, on the other [26]. Studies investigating load-dependent abnormal fatigability, maximal strength, subjective fatigue, and surface electromyography have shown a correlation between the changes in subjective fatigue and changes in strength in people with MS—but not a relationship between subjective fatigue and the changes in the amplitude of the electromyography. In other words, repetitive motor tasks did not lead to a performance decline, but fatigue feelings clearly increased during a repetitive motor task [27]. The increased experience of fatigue can be confounded by depression, and the objective signs of fatigability are normally not assessed in clinical praxis [19, 26, 28, 29]. Moreover, most published studies investigating MS-related fatigue assessed fatigue by questionnaires, as opposed to objective investigations.

As mentioned above, the MFIS can be divided into three subscales. The nine items of the “physical subscale” measure motor fatigue. Patients can perceive physical (motor) fatigue and mental (cognitive) fatigue simultaneously. An exact definition of motor fatigue is lacking, but usually, motor fatigue is defined as a reduction in maximal walking distance that cannot be explained by the degree of paresis, ataxia, or spasticity [30].

Gait recording using a camera system and infrared markers attached to the patient’s body and connected by cable to a unit worn on a belt allows the objective assessment of gait abnormalities after physical exertion and exhaustion. Therefore, the changes in gait parameters after physical exertion can be regarded as one possibility to distinguish objectively motor fatigue from fatigue by depression or normal tiredness—a clear diagnostic statement which, unfortunately, is lacking in the domain of cognitive fatigue and tiredness [30].

Studies using functional magnetic resonance imaging (fMRI) to test whether a different pattern of movement-associated cortical and subcortical activation might contribute to the development of fatigue in patients with MS showed more significant activation of the contralateral primary somatomotor cortex, the ipsilateral rolandic operculum, and the thalamus, indicating impaired interaction between functionally related cortical and subcortical areas in motor fatigue [31].

### Primary versus secondary fatigue

Studies investigating MS-related fatigue distinguish between primary and secondary fatigue. Stankoff et al. state that “in some cases, fatigue may be secondary to depression, cognitive dysfunction, poor sleep, or motor impairment. Nevertheless, for many patients, fatigue exists independently of motor weakness, cognitive, or mood disorders: this primary fatigue….” [16]. Yet the differentiation between primary fatigue (due to MS) and secondary fatigue (due to other diseases, e.g., depression and sleep disorders) is not always clear. The prevalence of restless legs syndrome (RLS) is four times higher in MS patients than in the general population and may be related to spinal cord damage [32, 33]. So, are fatigued MS patients with RLS due to spinal cord lesions suffering from *primary* or *secondary* fatigue? What exactly is *primary* fatigue?

### Discrimination between fatigue, motor fatigue, tiredness, and sleepiness

**Fig. 1:**
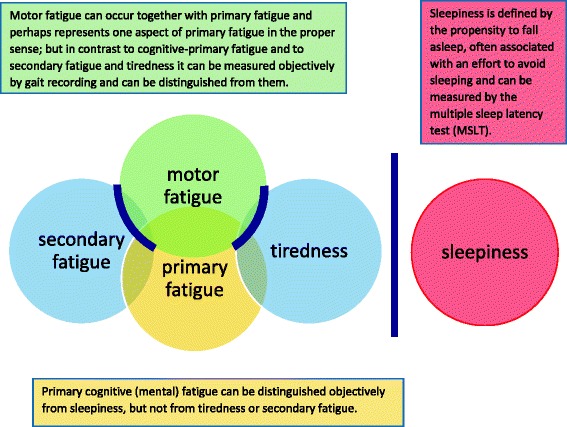
Distinction between primary fatigue, secondary fatigue, and sleepiness. This figure displays possibilities for distinguishing the primary fatigue from secondary fatigue, tiredness, and sleepiness. Whereas primary motor fatigue can be distinguished objectively from tiredness and secondary fatigue by gait recording, primary cognitive fatigue indeed cannot be distinguished objectively from tiredness and secondary fatigue. Sleepiness can clearly be distinguished from primary and secondary fatigue and motor fatigue and tiredness—as sleepiness can be measured objectively by the multiple sleep latency test

Figure 1 displays schematically the relationship between primary and secondary fatigue, tiredness, motor fatigue, and sleepiness. Whereas motor fatigue (as one aspect and a specific manifestation of primary fatigue) can be measured objectively by gait recording, mental (cognitive) primary fatigue cannot be distinguished objectively from tiredness and secondary fatigue. Sleepiness is completely different from primary or secondary fatigue or tiredness and can be measured objectively by multiple sleep latency test (MSLT) [20].

## Clinical correlates and biological mechanisms

### Disease course and disability

Patients with higher disability (according to the Expanded Disability Status Scale (EDSS)) [34] suffer from fatigue more frequently [35, 36], and older MS patients as well as those with longer disease duration tend to have more fatigue [36, 37]. Findings vary regarding disease course: Whereas some studies found fatigue to be more prevalent in progressive MS [17, 36, 37], other studies were not able to confirm this relationship [35, 38]. A recent study identified fatigue as an independent predictor of a subsequent MS diagnosis in a cohort of over 100 patients with a clinically isolated syndrome [39].

### Cognitive function, neuropsychological deficits, depression, and fatigue

MS is a complex neurological illness that is often accompanied by neuropsychiatric symptoms. Indeed, major depression is the most common comorbidity in MS [40]. In addition, more than 60% of MS patients experience cognitive impairment during the course of their illness, with information processing, executive functioning, attention, and memory most commonly affected [41–47]. Rather than broad cognitive impairment, MS is better characterized by relatively specific, domain-dependent deficits [48]. However, accurate neuropsychological assessment is complicated in MS, because fatigue and depression themselves may have detrimental effects on cognitive function. For instance, poor performance could be confounded by comorbid depressive symptomatology or fatigue, especially as cognitive complaints can be subtle. For example, the Paced Auditory Serial Addition Test (PASAT), often used as a primary tool to assess working memory and processing speed [49], may be vulnerable in repeated neuropsychological assessments to practice effects that obscure the impact of fatigue. More specifically, one study found that while PASAT performance increased with each trial, so did fatigue [50].

Similarly, while subjective fatigue has been associated with reduced cognitive performance [4, 51, 52], this relationship was not confirmed in other studies [12, 53–56], indicating that the interplay between cognitive dysfunction and fatigue is more complex than could be expected from direct adverse effects of fatigue on quality of life or disability. Indeed, it has been suggested that while cognitive exhaustion may heighten levels of fatigue, severe fatigue does not necessarily reduce cognitive performance [52].

In line with the well-known effects of depression in healthy subjects, and despite earlier negative findings (e.g., [57]), a review has corroborated the association between depression and cognitive function in MS [58]. More specifically, it was shown that high levels of depression mainly affect cognitive functions that are resource intensive, such as attention, working memory, or planning [59–61]. Notably, the adverse effects of depression may be mediated by reductions in processing speed [62], which have also been observed in non-depressed patients with MS [63–65].

An international consortium of MS experts has recommended a battery of tests known as the “Minimal Assessment of Cognitive Function in Multiple Sclerosis” (MACFIMS) for routine monitoring of MS-associated cognitive impairment [66]. The MACFIMS takes roughly 90 min to administer and assesses language (Controlled Oral Word Association Test), spatial processing (Judgment of Line Orientation Test), learning and memory (California Verbal Learning Test (CVLT)), Brief Visuospatial Memory Test (BVMT), processing speed, and working memory (Symbol Digit Modalities Test, PASAT) as well as executive function (Delis-Kaplan Executive Function System Sorting Test) [66]. Alternatively, an abbreviated combination is available that takes approximately 15 min to complete (Brief International Cognitive Assessment for Multiple Sclerosis (BICAMS)) and includes the SDMT, CVLT-II (first five recall trials only), and BVMT-R (first three recall trials only) [67].

In summary, assessment of neuropsychological impairment in MS patients needs to factor in comorbid depression and fatigue. Particularly, cognitive tests that rely predominantly on processing speed may be vulnerable to bias.

### Fatigue and sleep disorders

The Epworth Sleepiness Scale (ESS) is a screening tool that assesses sleepiness [22], but normal values do not entirely rule out sleep disorders as these are not always associated with sleepiness. Two polysomnographic studies investigating sleep disorders and fatigue in consecutive MS patients have demonstrated a significant relationship between fatigue and sleep disorders [17, 68]. Interestingly, a significant association existed between the presence of a sleep disorder and MFIS and FSS values, but not between fatigue and ESS values [17]. Both former groups monitored their individual study subjects in an open follow-up study and found that sleep medical treatment significantly reduced fatigue; however, prospective controlled studies are lacking [69, 70]. In the study by Veauthier et al., 49 out of 66 consecutive MS patients suffered from sleep disorders as confirmed by polysomnography [17]. The most common sleep disorders diagnosed in MS are obstructive sleep apnea (OSA), insomnia, periodic limb movement disorder (PLMD), restless legs syndrome (RLS), and sleep disruption due to nocturia.

Well-established diagnostic criteria that can accurately compare the prevalence of insomnia in the general population compared to MS patients are lacking. A discussion of insomnia in MS is provided in a recent review [2]. Importantly, in the clinical setting, over-the-counter drugs against insomnia (for example, products that contain diphenhydramine) can lead to next-day fatigue [71–73].

As mentioned above, prevalence of RLS is four times higher in MS compared to the general population. MS patients with RLS also show higher EDSS values and significantly reduced cervical cord fractional anisotropy in MRI examinations compared with MS patients without RLS—indicating a more pronounced cervical cord damage in MS patients with RLS [32, 33, 74, 75]. MS patients with more than ten periodic leg movements (PLMs) per hour of REM sleep showed a significantly higher disability measured by the EDSS [33].

With regards to MS and sleep apnea, it is currently unknown whether MS lesions can cause sleep apnea and whether the prevalence of sleep apnea is increased in MS [2, 76]. In the general population, sleep apnea can cause depression, and conversely, the treatment of sleep apnea by continuous positive airway therapy (CPAP) can be effective in the treatment of depression [77, 78]. This is especially important given the link between fatigue and depression in MS [19, 79]. Studies investigating the impact of treatment for nocturia in MS fatigue are lacking, but only half of MS patients with moderate to severe overactive bladder symptoms are treated with an anticholinergic medication [80].

As polysomnographic assessment is time consuming, defining which patients should undergo the test is critical. In order to develop a screening instrument, we performed a retrospective receiver operating characteristic (ROC) analysis of the investigated MS patients investigated by polysomnography (PSG)—using the MFIS for daytime dysfunction and the Pittsburgh Sleep Quality Index (PSQI) for sleep disturbances [81, 82]. By jointly applying the MFIS cutoff of 34 and the PSQI cutoff of 5 (either MFIS > 34 or PSQI > 5), we achieved a sensitivity of 89.8% for sleep disorders (specificity 58.8%, positive predictive value 86.3%, negative predictive value 66.7%).

### Structural and functional MRI correlates

As structural and diffuse brain damage are detectable from the earliest disease stages, multiple studies have attempted to relate fatigue severity assessed by different questionnaires and sometimes in conjunction with cognitive dysfunction to various neuroimaging findings acquired using structural, functional, and metabolic imaging modalities [83–88]. For example, a magnetic resonance spectroscopy study has indicated decreased N-acetylaspartate-creatine ratios (NAA/Cr) as a marker of axonal metabolic integrity with increasing fatigue scores, suggesting diffuse and widespread axonal dysfunction as a contributor to fatigue in MS [89]. Structural imaging studies focusing on the quantification of atrophy have reported an association of fatigue severity with atrophy in the thalamus and a number of different cortical gray matter regions (superior frontal and inferior parietal gyrus, parietal lobe) in patients with relapsing-remitting MS [90, 91]. Yaldizli et al. found an association between callosal atrophy and fatigue and cognitive performance in a large cohort of MS patients with various disease courses [92], and Nygaard et al. reported a relation between regionally smaller cortical surface areas and volumes and higher depression and fatigue scores in relapsing-remitting MS patients [93]. Although Gobbi et al. did not find an association between white matter atrophy and lesion distribution [94], a previous study by Sepulcre et al. suggested a correlation between T2 lesion load and fatigue severity and between fatigue scores and gray matter atrophy in frontal regions [95]. Damasceno et al. found an association between fatigue severity and atrophy in various cortical and gray matter regions; however, after controlling for disability and depression, only caudate and accumbens volumes remained statistically significant (interestingly, the nucleus accumbens is an important mesolimbic structure involved in motivational and reward processes) [96]. Caudate and basal ganglia were also identified as regions related to clinical fatigue by other imaging modalities. A positron emission tomography study by Roelcke et al. found reduced glucose metabolism in the frontal cortex and basal ganglia in MS patients with fatigue [97], and in a resting-state fMRI study, Finke et al. showed that fatigue severity was negatively correlated with functional connectivity of the basal ganglia with the medial prefrontal cortex, precuneus, and posterior cingulate cortex [98]. These connectivity changes suggest—in line with the above mentioned study by Damasceno et al. [96]—that impairment of both motor and non-motor functions of the basal ganglia, which also include reward processing and motivation, is involved in the pathophysiology of fatigue in MS. Another fMRI study applying a motor task by Rocca et al. found decreased activation of various frontal and temporal cortical regions and—importantly—the basal ganglia in MS patients with fatigue, versus MS patients without fatigue and healthy controls. The authors interpreted this as a maladaptation of motor network recruitment and a central contributor to fatigue [99].

Although these and other imaging studies provide interesting data on imaging correlates of fatigue and underscore that dopaminergic structures like the basal ganglia may be significantly related to the perception of fatigue, they merely describe statistical correlations or associations and do not shed light on causality. Moreover, comparability between studies is limited by the heterogeneity of the applied imaging modalities, the clinical cohorts assessed, and the fatigue questionnaires used, which likely at least in part accounts for the discrepancies between study findings. Moreover, in future studies, sleep disorders as a major cause for secondary fatigue and as an important confounder should be ruled out by polysomnography prior to study enrollment.

### Inflammation and neuroendocrine abnormalities

One intriguing observation from animal models and experimental studies in humans is that cytokines and other inflammatory stimuli can induce sickness behavior symptoms, including anorexia, hypoactivity, and hyperthermia, that resemble some aspects of fatigue [100–102]. This so-called sickness behavior has therefore been proposed as a pathobiological model of fatigue in the context of inflammatory disorders such as MS [103]. However, clinical studies applying this model in the specific context of MS have yielded mixed results. Circulating levels of several inflammatory markers such as interleukin-2 (IL-2), neopterin, ICAM-1 (intercellular adhesion molecule 1), and C-reactive protein (CRP) were not correlated with fatigue [104, 105]. In contrast, two studies examining proinflammatory cytokines such as TNFα (tumor necrosis factor alpha) (but not interferon-γ (IFNγ)) mRNA in lymphocytes [106] and TNFα and IFNγ protein production after in vitro stimulation [107] reported positive correlations with fatigue. A study subsequent to the latter also found increased intracellular in vitro production of IFNγ that correlated fatigue [108]. These cytokines did not yield significant associations with fatigue scores in a more comprehensive serum cytokine screen, which however suggested an association of fatigue with interleukin-6 (IL-6) [109].

Beyond inflammatory markers, several studies have also investigated the relationship between alterations in neuroendocrine systems, such as the hypothalamo-pituitary-adrenal (HPA) axis, and MS-related fatigue. Again, study findings were mixed. Whereas Gottschalk et al. found a higher activity of the HPA axis in MS fatigue patients, as evidenced by significantly increased adrenocorticotropin (ACTH) concentrations, Heesen et al. reported that HPA-axis feedback-regulation (as determined by the Dex-CRH suppression test) axis activity was not related to fatigue [107, 110]. Resting levels of cortisol (as measured by a single midday blood sample) were also not associated with fatigue in another study [111]. More recently, one study reported an association of fatigue in relapsing-remitting MS (RR-MS) with lower waking cortisol levels and an increased response to awakening (cortisol awakening response (CAR)), indicating that cortisol could play a role in the perception of fatigue [112], but two studies assessing the circadian slope of cortisol secretion did not find an association with MS fatigue [108, 112].

In summary, while the role of inflammation in the pathobiology of “fatigue-like” symptoms is well supported by animal models and experimental studies in humans, no individual immune or neuroendocrine markers have consistently been associated with fatigue in MS. Some of this might be due to the large heterogeneity of markers, challenge paradigms, and the definition of fatigue used in each particular study. Moreover, in light of the abovementioned high prevalence of sleep disorders in MS fatigue, future studies investigating the neuroinflammatory and neuroendocrine aspects of MS-associated fatigue should take care to apply polysomnography to exclude possible bias resulting from sleep disorders as confounder.

### Methodical limitations and further research direction

Diversity between study cohorts (e.g., with or without secondary progression, level of disability, treatment naïve or not) is a long-standing problem hampering definitive interpretation of research into MS as a whole. The investigation of MS-related fatigue is additionally complicated by a lack of standardization between fatigue scales and, even more seriously, by the fact that the potential confounders (sleep disorders and drug side effects) are not excluded as a matter of course. Definitively identifying mutual relationships between distinct parameters (imaging or depression or disease course or cognitive dysfunction or sleep disorders or inflammation and fatigue) across different studies becomes neigh on impossible. To date, not one study into MS-related fatigue has examined all parameters and excluded all known confounders. Such a task would be even more difficult in the context of urgently needed large multicenter studies.

## Treatment of MS fatigue

### Immune therapy and fatigue

The same methodical limitations apply to the published data on the impact of MS treatment on fatigue. The lack of studies investigating the influence of existing MS therapies on MS fatigue as a primary endpoint and the influence of other confounders, such as age, disease course and duration, questionnaires used, medication, comorbid disorders (e.g., depression), and the lack of a control group in most studies, have hampered insight into the effect of disease-modifying therapies on MS-related fatigue. However, a number of studies suggest that beta interferons and glatiramer acetate (GA) reduce fatigue (with GA treatment potentially more effective) [113–116]. The effect of natalizumab on fatigue varied between studies [117, 118]. Whereas Penner et al. found that fatigue levels remained constant or decreased in the majority of patients after 1 year of natalizumab treatment, Kunkel et al. found no significant changes, although this may have been due to the study’s small cohort size [117, 118].

Some limited results show that fingolimod might improve fatigue after changing from beta interferon to fingolimod (but not from GA) [119, 120]. However, no evidence exists that teriflunomide, dimethyl fumarate, or alemtuzumab improve fatigue [121–123]. In fact, one study identified fatigue as a side effect of dimethyl fumarate treatment [122].

### Management strategies recommended by the MS Council

As fatigue is not specific or unique to MS [124–126], in 1998, the MS Council for Clinical Practice Guidelines recommended performing blood tests and evaluating comorbid medical conditions, iatrogenic factors, depression or psychological distress, sleep disturbances, and, where applicable, as next diagnostic step sleep studies (polysomnography and multiple sclerosis latency test) [9]. Chronic obstructive pulmonary disease (COPD) should also be excluded [9]. After ruling out or addressing any comorbid medical conditions, drug therapy, (aerobic) exercise, and, among others, self-management strategies were recommended. Self-management strategies included activities such as cessation of smoking, modifying dietary habits, optimizing time management, adjusting activity levels, taking naps, consumption of cool beverages, cool showers and baths for heat intolerance, exercise programs, or engaging in relaxation exercises.

### Drug therapy

One recently published trial (alfacalcidol versus placebo) showed a significant reduction of fatigue under therapy with alfacalcidol (an active vitamin D3 metabolite requiring activation in the liver) [127]. Further studies are needed in order to confirm this effect. Apart from vitamin D, in recent studies, modafinil, amantadine, carnitine, or pemoline [16, 128–136] have generally not been found to be effective in treating MS-related fatigue. Our review of the literature found six meta-analyses of the randomized controlled trials (modafinil, amantadine, carnitine, and pemoline) [16, 128, 130, 131, 133–135, 137–140]. Whereas one meta-analysis investigating three studies published before 1996 found a significant effect of amantadine superior to placebo [141], five other meta-analyses analyzing these three studies and further recent studies did not report any significant effect of the investigated drugs [129, 136, 142–144]. One study, however, did find a significant improvement of fatigue under treatment with carnitine compared to amantadine [140]. Aspirin was found to be an effective treatment for fatigue in another study [145], although this was countered by a subsequent study comparing aspirin with amantadine treatment [130].

### Motor fatigue and fampridine

4-Aminopyridine (dalfampridine, fampridine), a potassium channel blocker, is approved by the US Food and Drug Administration, Health Canada, and the European Medicines Agency as a treatment for MS-related walking ability [146]. Clinical trials have shown improvement in walking speed compared to placebo (the percentage of MS patients with an improvement in 6-min walk test of >20% (45.1%) was significantly greater with dalfampridine relative to placebo (14.3%)) [146, 147].

Furthermore, two studies showed that fampridine improves MS fatigue as assessed using the visual analog scale (VAS), the MFIS, and the FSS [148, 149]. Treatment with fampridine should be stopped immediately in patients presenting with hypersensitivity symptoms, such as swollen face, mouth, lips, throat or tongue, reddening or itching of the skin, chest tightness and breathing problems, and seizures (up to 1 in 100 people). A number of other unpleasant but harmless side effects exist, including urinary tract infection (>10%) and dizziness, headache, sleep disturbances, and anxiety (<10%). Fampridine is contraindicated in patients with a creatinine clearance lower than 80 ml/min, and therefore, renal function should be monitored. Initial prescription should be limited to 2 weeks of therapy, which is the duration needed to assess clinical benefit. Extension of treatment should be subject to improvement as assessed by a timed walking test. If no improvement is observed at this point, the fampridine treatment should be discontinued. Fampridine is contraindicated during pregnancy.

### Fatigue management interventions, rehabilitation intervention and cognitive behavioral therapy

Rehabilitation (exercise or physical therapy, educational interventions such as mindfulness training, self-management program, vestibular rehabilitation) and cognitive behavioral therapy (CBT) are significantly effective against MS-related fatigue [136, 150–153]. Moreover, internet-based CBT has been investigated and could play a greater role in the future [154, 155].

A 2014 systematic review and meta-analysis by Asano and Finlayson compared pharmacotherapy, exercise, and educational interventions for MS-related fatigue [136], suggesting that the eight reports of educational approaches yielded a significant overall effect with an effect size of 0.54 (95% CI 0.30–0.77, *p* < 0.001). In particular, the three trials that used CBT exhibited the largest effect within the educational intervention subsection of the review [154–156]. One additional trial has been published since the above review combining CBT with energy conservation. Here, too, significantly reduced fatigue was found (standardized effect size 0.54). A follow-up analysis found that some of the effects were maintained at 12 months post-trial [157].

Overall, strong evidence exists that educational interventions have a definitive moderate ability to reduce MS-related fatigue. It should be noted that the studies surveyed were generally combined elements of CBT, mindfulness, skill training, and educational components. Published evidence to date suggests that CBT-based interventions are the most effective elements of a fatigue management program (see Table 1). Finally, the settings of these interventions also vary from group to one-on-one sessions or from in-person to telephone or internet intervention. The available trials (see Table 1) suggest that individual intervention might yield larger effects than group interventions; however, this has not been formally studied to date. For the time being, the setting used should be based on the resources available and the specific needs and requirements of the patient population. Remote access options like telephone or Internet sessions might be particularly suitable for patients with mobility problems or patients in rural areas.

**Table 1:** Trials using educational interventions in MS-related fatigue

	Sample size	Intervention	Effect size (according to meta-analysis by Asano and Finlayson)
Mohr et al. 2003 [156]	60	CBT (individual sessions)	0.80 (0.19–1.42)
Mathiowetz et al. 2005 [188]	169	Fatigue management program (energy conservation course) (group sessions)	0.42 (0.08–0.76)
Kos et al. 2007 [189]	51	Multidisciplinary fatigue management program (group sessions)	−0.16 (−0.72 to 0.38)
Van Kessel et al. 2008 [155]	72	CBT (individual and phone sessions)	0.99 (0.50–1.48)
Grossman et al. 2010 [150]	150	Mindfulness (group sessions)	0.42 (0.09–0.74)
Hugos et al. 2010 [190]	30	Fatigue management program (group sessions)	0.43 (−0.29 to 1.57)
Finlayson et al. 2011 [191]	190	Fatigue management program (phone sessions)	0.53 (0.19–0.86)
Moss-Morris et al. 2012 [154]	40	CBT (online + phone sessions)	1.11 (0.43–1.78)
Thomas et al. 2013 [157]	164	CBT + energy effectiveness training (group)	0.54 (not included in meta-analysis)

### Treatment of underlying sleep disorders

The first step to addressing MS-related fatigue is diagnosing and treating any underlying sleep disorders. Two studies of sleep disorders and fatigue in consecutive MS patients have demonstrated a significant relationship between fatigue and sleep disorders [17, 68]. In the study by Veauthier et al., 96% of fatigued MS patients were suffering from a sleep disorder [17]. Sleep disorders are frequent in MS and have been significantly associated with higher MFIS values. MS patients suffering from sleep disorders show increased MFIS values compared to MS patients who are not suffering from sleep disorders (SD ±42.8 versus ±20.5, *p* < 0.001) [17]. Besides depression and disability, sleep disturbances decrease health-related quality of life (HRQoL) [158, 159]. Although OSA is a prevalent pathological condition and represents an increased risk for cardiovascular diseases, a majority of patients remain undiagnosed [160, 161]. In particular, MS patients suffering from comorbid obstructive sleep apnea (OSA) or insomnia showed significantly decreased HRQoL for the “sleep” area (sleep subscale). But furthermore, OSA and insomnia patients show increased values in the “energy” and “emotional” subscales as well indicating that sleep disorders lead to impaired daytime functioning (and in OSA patients also for “physical abilities”) [158].

The most important sleep disorders in this context are OSA, insomnia, periodic limb movement disorder (PLMD), restless legs syndrome (RLS), and nocturia [2]. In two open follow-up studies, the treatment of an underlying sleep disorder (continuous positive airway pressure (CPAP) for OSA, drug therapy and CBT for insomnia, and drug therapy for RLS and PLMD) significantly reduced fatigue, but prospective controlled studies are lacking [69, 70].

Many studies investigating fatigue have used the Epworth Sleepiness Scale (ESS) as a screening tool for sleep disorders [16, 22, 162]. That is not entirely correct. The ESS is a diagnostic instrument for sleepiness, not fatigue, and is especially recommended in the context of hypersomnia, OSA, and narcolepsy. A part of patients suffering from sleep disorders feel sleepy, but a significant proportion does not feel sleepy but rather fatigued. Therefore, the ESS is not a suitable questionnaire for all sleep disorders. In insomnia and other sleep disorders like RLS or PLMD, specific questionnaires are recommended: the Pittsburgh Sleep Quality Index (PSQI) or the Insomnia Severity Index (ISI) for insomnia and the RLS Diagnostic Index (RLS-DI) for RLS [81, 163, 164]. These questionnaires cannot reflect the complex symptoms of sleep disorders and are only screening tools—clinical interview by a sleep expert and objective testing (polysomnography) are the gold standard of sleep medical diagnosis.

### The Berlin Treatment Algorithm

**Fig. 2:**
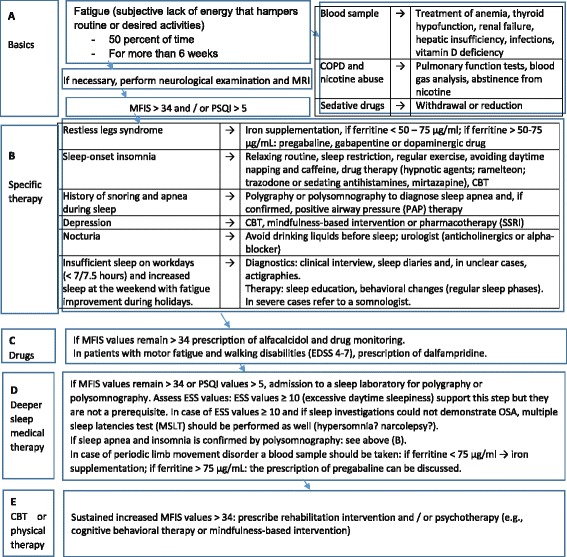
The Berlin Treatment Algorithm. Please note: Restless legs syndrome can be diagnosed on the basis of the following four minimal criteria: an urge to move the legs (usually accompanied by uncomfortable sensations), which begin or worsen during rest and are relieved by movement predominantly in the evening or night. Depression can be diagnosed by structured interviews. Self-report scales (e.g., Beck Depression Inventory (BDI)) can be useful to screen for depression. Abbreviations: *MFIS* Modified Fatigue Impact Scale, *PSQI* Pittsburgh Sleep Quality Index, *ESS* Epworth Sleepiness Scale, *COPD* chronic obstructive pulmonary disease, *SSRIs* selective serotonin reuptake inhibitors, *CBT* cognitive behavioral therapy

It is one thing to recommend the treatment of underlying sleep disorders in this issue in general, but another to say which patient has to be sent to the sleep specialist or sleep laboratory. Because of the complexity of the issue and due to the fact that clinicians need some practical instructions, the discussion of the different treatment options does not replace the need for a practical guide with specific procedures and measures. We would like to take the opportunity to introduce our Berlin Treatment Algorithm of MS fatigue, which we developed in the last years. Figure 2 outlines the treatment algorithm.

#### Phase A: basic recommendations

The Berlin Treatment Algorithm follows the recommendations of the MS Council for Clinical Practice Guidelines as a first step: As an initial step, blood tests should be performed to rule out anemia, thyroid dysfunction, renal and hepatic insufficiency, vitamin D deficiency, and infections. In cases of possible COPD (due to smoking), pulmonary function tests and blood gas analysis are also recommended. At this stage, medical history questionnaires should also address a patient’s use of sedative drugs, including over-the-counter medicines, which might worsen fatigue. Weaning or reducing patient consumption of such drugs would be the next necessary step. If the patient has recently been suffering from fatigue, a neurological examination should be performed to rule out relapse or disease progression and an MRI should be performed in order to assess radiographic disease activity [165]. Once the above confounders have either been addressed or excluded, the patient should fill out the MFIS and the PSQI. Then, all patients with higher MFIS values >34 and/or PSQI values >5 should enter the next phase.

#### Phase B: specific therapy

At this stage, possible causes of fatigue should be identified by a diagnostic interview. The latter should include questions about both current and past fatigue symptoms and focus mainly on determining whether nocturia, depression, and sleep disorders (OSA, insomnia, RLS, behaviorally induced insufficient sleep syndrome (BIISS)) play a role [21].

##### Restless legs syndrome

RLS is defined by an urge to move the legs, usually accompanied by uncomfortable and unpleasant sensations in the legs, which is partially or totally relieved by movement. The discomfort begins or worsens during periods of rest or inactivity and in the evening or at night on condition that its occurrence is not solely accounted for as symptoms primary to another medical or a behavioral condition [166]. RLS is often misdiagnosed in MS patients, and RLS-associated symptoms are often misinterpreted as spasticity in more disabled patients.

Treatment of RLS symptoms consists of iron supplementation, if ferritin serum levels are lower than 75 mcg/l, and dopaminergic drugs [167–169]. Pregabalin is effective against RLS symptoms as well, but it is not approved in some countries for the treatment of RLS [170].

The questionnaire *Restless Legs Syndrome Diagnostic Index* (RLS-DI) is used as a screening tool; however, this cannot replace a diagnostic interview by a sleep expert. The International RLS Study Group (IRLSSG) rating scale should be used for the assessment of the response to therapy [164, 171].

##### Insomnia

Whereas impaired sleep maintenance can be an unspecific symptom of sleep disorders in general (OSA, insomnia, and others), delayed sleep onset with prolonged sleep latencies before falling asleep is a clear indication for sleep-onset insomnia. Such patients can be treated with sleep restriction therapy and sleep hygiene education, relaxing routine within an hour before bedtime, regular exercise, avoiding daytime napping, and late heavy meals [172, 173]. Sleep restriction is an easy and very efficacious therapy against sleep-onset insomnia [174]. Prescription and over-the-counter drug therapies include short-acting non-benzodiazepine sedative-hypnotic agents; ramelteon, a melatonin receptor agonist; trazodone; antidepressants; and sedating antihistamines [175]. Thyroid hyperfunction should be ruled out, and coffee should be avoided (in particular, in the afternoon and evening).

##### Behaviorally induced insufficient sleep syndrome

Sleepiness may be caused by behaviorally induced insufficient sleep syndrome (BIISS), which can only be diagnosed by a detailed medical history [21]. Doctors should not only ask about sleep problems, but also about time spent in bed (currently and in the past, on workdays, and at weekends) and about fatigue severity during workdays and during holidays. Longer sleep times on the weekend or during holidays point to BIISS. Sleep diaries or actigraphy may be helpful. In severe cases, referral to a sleep expert can be necessary [167].

##### Obstructive sleep apnea

If patients suffer from sleepiness (especially in case of ESS values ≥10), they should be referred to a sleep specialist or admitted to a sleep laboratory, for assessment of possible narcolepsy, hypersomnia, or (more frequently) obstructive sleep apnea; symptoms of the latter include snoring, non-restorative sleep, morning headaches, dry mouth, nocturnal gasping or apnea, cardiovascular diseases, increased neck circumference, obesity, and a family history of OSA [20].

##### Nocturia

Patients with nocturia (≥2 times per night) should be referred to a urologist, and a treatment with an anticholinergic or alpha-blocker should be considered [80, 176].

##### Depression

Depression should be diagnosed using current criteria (e.g., Diagnostic and Statistical Manual of Mental Disorders, Fifth Edition (DSM-V) [177] or the International Statistical Classification of Diseases and Related Health Problems (ICD-10) [178]). This should ideally be done using standardized tools such as structured diagnostic interviews by experienced clinicians. Self-report scales such as the Beck Depression Inventory (BDI-II) can be used as a screening tool to identify patients requiring further diagnostic work-up. Recommended cutoffs for the general population are BDI values >13 in treated depressed patients and BDI-II >21 in treatment naïve patients [179]. For MS patients, these cutoffs might need to be adjusted to optimize sensitivity and specificity, depending on the clinical context [13, 180]. As a brief screening tool, the two-item approach by Mohr and colleagues has been recommended by the American Academy of Neurology (AAN) task force [156, 181].

In cases of depression, the treatment should include tailored treatment algorithms and multimodal approaches and a comprehensive treatment should include primarily psychotherapy and optionally pharmacotherapy [182]. Selective serotonin reuptake inhibitors (SSRIs) are generally considered to be an effective and well-tolerated first-line treatment [183]. The guidelines published by the AAN did not recommend antidepressant pharmacotherapy for depression in MS due to a lack of conclusive evidence; however, more recently, a meta-analysis reported overall beneficial effects [181, 184, 185]. Cognitive behavioral therapy and mindfulness-based interventions have shown efficacy in improving depressive disorders in MS [181, 185].

#### Phase C: drug therapy

Fampridine prescribed for walking ability may be effective against fatigue as well, and one recent published randomized controlled trial (RCT) showed a substantial improvement of fatigue under therapy with vitamin D (alfacalcidol, if blood calcium levels are not higher than 10.5 mg/dl)—but despite the substantial psychosocial burden of MS-related fatigue, there are no pharmacological treatments formally approved by regulatory authorities [127, 129, 136, 149, 186]. Despite poor evidence, individual attempts to improve fatigue with modafinil, amantadine or carnitine may be undertaken. 

#### Phase D: intensified sleep medical therapy

Patients without improvement in fatigue following measures undertaken during the preceding phases should be admitted to a sleep laboratory in order to diagnose or exclude other underlying sleep disorders according to the ICSD-3 (International Classification of Sleep Disorders, Third Edition), which cannot be diagnosed by the diagnostic interview and questionnaires alone (for example, sleep-related breathing disorders including central apnea, obstructive sleep apnea, obesity hypoventilation syndrome, periodic limb movement disorder, parasomnia, insomnia) [20]. In some cases, a specified sleep medical treatment (continuous positive airway pressure (CPAP) or drug therapy) can be started and fatigue may improve.

#### Phase E: rehabilitation and cognitive behavioral therapy

If fatigue persists, patients should participate in exercise therapy (aerobic exercise or resistance training) at least once a week or CBT [9, 183]. This can be undertaken individually or as part of a group therapy and would be facilitated by tertiary MS centers collaborating with physical therapists or physical medicine and rehabilitation specialists in order to develop interned-based and cost-effective interventions.

## Outlook

In the future, novel approaches such as smartphone–based applications might play a role in measuring performance and patient-reported outcomes and improving fatigue by self-management strategies [187].

## Conclusions

The treatment of MS-related fatigue remains a challenging task, particularly due to the lack of a proven pharmacological therapy. However, many other therapeutic strategies have been shown to be effective in the treatment of MS-related fatigue. Therefore, a comprehensive approach including self-management strategies, rehabilitation, CBT, and the treatment of underlying sleep disorders and potentially other comorbidities is reasonable. The aim of the algorithm presented is to provide the treating physicians with a practical guide facilitating diagnosis and treatment of MS fatigue in individual patients.

### Expert recommendation

Personalized medicine is a promising new therapeutic strategy in order to treat MS-related fatigue. The treatment of fatigue should be tailored to the MS patients—taking into account comorbid disorders such as RLS, insomnia, depression, history of snoring and sleep apnea, nocturia, and sleep deprivation as well as side effects of the medication. Patients with high MFIS or PSQI values indicating a high risk of sleep disorders should be investigated by polygraphy or polysomnography.

## Abbreviations

AAN: American Academy of Neurology
ACTH: Adrenocorticotropic hormone
BDI-II: Beck Depression Inventory
BICAMS: Brief International Cognitive Assessment for Multiple Sclerosis
BIISS: Behaviorally induced insufficient sleep syndrome
BVMT: Brief Visuospatial Memory Test
CAR: Cortisol awakening response
CBT: Cognitive behavioral therapy
COPD: Chronic obstructive pulmonary disease
CPAP: Continuous positive airway pressure
CRP: C-reactive protein
CVLT: California Verbal Learning Test
DSM-V: Diagnostic and Statistical Manual of Mental Disorders, Fifth Edition
EDSS: Expanded Disability Status Scale
ESS: Epworth Sleepiness Scale
FIS: Fatigue Impact Scale
fMRI: Functional magnetic resonance imaging
FSMC: Fatigue Scale for Motor and Cognitive Functions
FSS: Fatigue Severity Scale
GA: Glatiramer acetate
HPA: Hypothalamo-pituitary-adrenal
HRQoL: Health-related quality of life
ICAM-1: Intercellular adhesion molecule 1
ICD-10: International Statistical Classification of Diseases and Related Health Problems
ICSD-3: International Classification of Sleep Disorders, Third Edition
IFNγ: Interferon-γ
IL-2/IL-6: Interleukin-2/-6
MACFIMS: Minimal Assessment of Cognitive Function in Multiple Sclerosis
MFIS: Modified Fatigue Impact Scale
MS: Multiple sclerosis
MSLT: Multiple sleep latency test
MSQLI: Multiple Sclerosis Quality-of-Life Inventory
NAA/Cr: N-acetylaspartate-creatine ratio
OSA: Obstructive sleep apnea
PASAT: Paced Auditory Serial Addition Test
PLMD: Periodic limb movement disorder
PSG: Polysomnography
PSQI: Pittsburgh Sleep Quality Index
RCT: Randomized controlled trial
RLS: Restless legs syndrome
RLS-DI: RLS Diagnostic Index
ROC: Receiver operating characteristic
RR-MS: Relapsing-remitting MS
SSRIs: Selective serotonin reuptake inhibitors
TNFα: Tumor necrosis factor alpha
